# Circulating insulin-like growth factor axis and the risk of pancreatic cancer in four prospective cohorts

**DOI:** 10.1038/sj.bjc.6603826

**Published:** 2007-05-29

**Authors:** B M Wolpin, D S Michaud, E L Giovannucci, E S Schernhammer, M J Stampfer, J E Manson, B B Cochrane, T E Rohan, J Ma, M N Pollak, C S Fuchs

**Affiliations:** 1 Department of Medical Oncology, Dana-Farber Cancer Institute, Boston, MA, USA; 2 Department of Medicine, Brigham and Women's Hospital, Harvard Medical School, Boston, MA, USA; 3 Department of Epidemiology, Harvard School of Public Health, Boston, MA, USA; 4 Department of Nutrition, Harvard School of Public Health, Boston, MA, USA; 5 Channing Laboratory, Department of Medicine, Brigham and Women's Hospital, and Harvard Medical School, Boston, MA, USA; 6 Ludwig Boltzmann-Institute for Applied Cancer Research, KFJ-Spital, Vienna, Austria and Applied Cancer Research – Institute for Translational Research Vienna (ACR–ITR VIEnna), Vienna, Austria; 7 Division of Preventive Medicine, Brigham and Women's Hospital, Boston, MA, USA; 8 Division of Public Health Sciences, University of Washington School of Nursing, Seattle, WA, USA; 9 Department of Epidemiology and Population Health, Albert Einstein College of Medicine, Bronx, NY, USA; 10 Department of Medicine and Oncology, Jewish General Hospital and McGill University, Montreal, Quebec, Canada

**Keywords:** pancreatic cancer, insulin-like growth factor, insulin-like growth factor binding protein, insulin

## Abstract

Insulin-like growth factor (IGF)-I induces growth in pancreatic cancer cells and blockade of the IGF-I receptor has antitumour activity. The association of plasma IGF-I and IGF binding protein-3 (IGFBP-3) with pancreatic cancer risk has been investigated in two small studies, with conflicting results. We conducted a nested case–control study within four large, prospective cohorts to investigate whether prediagnostic plasma levels of IGF-I, IGF-II, and IGFBP-3 were associated with pancreatic cancer risk. Plasma levels in 212 cases and 635 matched controls were compared by conditional logistic regression, with adjustment for other known pancreatic cancer risk factors. No association was observed between plasma levels of IGF-I, IGF-II, or IGFBP-3 and incident diagnosis of pancreatic cancer. Relative risks for the highest *vs* the lowest quartile of IGF-I, IGF-II, and IGFBP-3 were 0.94 (95% confidence interval (CI), 0.60–1.48), 0.96 (95% CI, 0.61–1.52), and 1.21 (95% CI, 0.75–1.92), respectively. The relative risk for the molar ratio of IGF-I and IGFBP-3, a surrogate measure for free IGF-I, was 0.84 (95% CI, 0.54–1.31). Additionally, no association was noted in stratified analyses or when requiring longer follow-up. In four prospective cohorts, we found no association between the risk of pancreatic cancer and prediagnostic plasma levels of IGF-I, IGF-II, or IGFBP-3.

The insulin-like growth factor (IGF) axis has been implicated in the development of several malignancies (Renehan *et al*, 2004). Insulin-like growth factor-I is a hormone and growth factor produced predominantly in the liver under the regulation of growth hormone, but also produced locally in multiple tissue types. Insulin-like growth factor-II is structurally similar to IGF-I, primarily produced in the liver, and maternally imprinted (Khandwala *et al*, 2000). More than 80% of circulating IGF-I and IGF-II are bound to IGF binding protein-3 (IGFBP-3), in a protein complex that is confined to the vascular compartment (Firth and Baxter, 2002). Tissue IGF bioactivity is thought to be determined by free IGF, the component of IGF not bound to IGF binding proteins, which binds to the IGF-I receptor (IGF-IR) on the target cell surface (Jones and Clemmons, 1995). Insulin-like growth factor-I and IGF-IR are highly expressed in pancreatic cancer cell lines, where initiation of intracellular signalling through IGF-IR leads to decreased apoptosis and increased proliferation, invasion, and expression of mediators of angiogenesis (Ohmura *et al*, 1990; Bergmann *et al*, 1995; Stoeltzing *et al*, 2003; Zeng *et al*, 2003; Neid *et al*, 2004).

High plasma levels of IGF-I and low levels of IGFBP-3 are associated with the development of prostate (Chan *et al*, 1998), colorectal (Ma *et al*, 1999), and premenopausal breast (Hankinson *et al*, 1998) cancer. In addition, loss of imprinting at the IGF-II locus leads to increased expression of IGF-II and higher rates of malignancy (Cui *et al*, 2003; Feinberg, 2004; Sakatani *et al*, 2005). Two small, nested case–control studies of IGF-I, IGFBP-3, and pancreatic cancer risk have yielded conflicting results (Lin *et al*, 2004; Stolzenberg-Solomon *et al*, 2004).

To further investigate whether plasma IGF-I, IGF-II, and IGFBP-3 levels are associated with the risk of pancreatic cancer, we performed a nested case–control study using the combined resources of four large prospective cohort studies, with blood samples collected before cancer diagnosis. We hypothesized that elevated levels of IGF-I and IGF-II and/or depressed levels of IGFBP-3 would predict an increased risk of pancreatic cancer.

## MATERIALS AND METHODS

### Subjects

Pancreatic cancer cases and matched controls through 2003 were drawn from four prospective cohort studies: the Nurses' Health Study (NHS), Health Professionals Follow-up Study (HPFS), Physicians' Health Study (PHS), and Women's Health Initiative (WHI). The NHS was initiated in 1976 when 121 701 US female registered nurses aged 30–55 responded to a mailed questionnaire regarding individual characteristics, habits, and medical history. The HPFS began in 1986 when 51 529 male health professionals aged 40–76 years responded to a similarly mailed questionnaire. The PHS was initiated in 1982 as a randomized clinical trial evaluating aspirin and *β*-carotene among 22 071 male physicians aged 40–84 years. The WHI observational component is a multi-centre cohort study of 93 676 postmenopausal women aged 50–79 years, enrolled between 1994 and 1998. In each of these cohort studies, regular follow-up questionnaires were mailed to participants every 1–2 years to update exposure data and medical history. Deaths among non-respondents were actively ascertained. All participants provided consent upon enrollment. The current study was approved by the Human Research Committee at Brigham and Women's Hospital, Boston, MA, USA.

If a participant (or next of kin for decedents) reported a diagnosis of pancreatic cancer, the diagnosis was confirmed by medical record and pathology report review. If the primary cause of death on a death certificate was a previously unreported or unconfirmed case of pancreatic cancer, family members were contacted for permission to retrieve medical records and confirm the diagnosis. Most deaths in these cohorts were reported by family members or by the postal service in response to follow-up questionnaires. In addition, searches of the National Death Index were conducted for non-responders; this method has a sensitivity of 98% or greater in identifying decedents (Rich-Edwards *et al*, 1994).

In each cohort study, blood samples drawn from participants were kept chilled until processing, separated into plasma, erythrocytes, and buffy coat, and stored as multiple aliquots in liquid nitrogen freezers. During storage, precautions were taken to ensure that no specimens thawed or warmed substantially. Blood was drawn from 18 225 men in the HPFS between 1993 and 1995, 32 826 women in the NHS from 1989 to 1990, 14 916 men in the PHS from 1982 to 1984, and 93 676 women in the WHI between 1994 and 1998.

Eligibility criteria for potential cases included no prior history of malignancy (other than non-melanoma skin cancer), available plasma sample, and two or more years between plasma collection and pancreatic cancer diagnosis. At the time of data set creation, the diagnosis of pancreatic cancer was confirmed by medical record review for all but four reported cases. Controls were required to have an available plasma sample and no cancer diagnosis (other than non-melanoma skin cancer). Three controls were matched to each case based on year of birth, smoking status (current, past, never), prospective cohort, month of blood draw, and fasting status at time of blood draw.

### Plasma assays of IGF-I, IGF-II, and IGFBP-3

Plasma levels of IGF-I, IGF-II, and IGFBP-3 were assayed in the laboratory of Dr Michael N Pollak (Jewish General Hospital and McGill University) by enzyme-linked immunosorbent assay with reagents from Diagnostic Systems Laboratory (Webster, TX, USA). Plasma samples from cases and matched control subjects were assayed in the same batch to minimize interassay variability, and quality control samples were inserted randomly. Laboratory personnel were unable to distinguish among case, control, and quality control samples. The mean intra-assay coefficients of variation for IGF-I, IGF-II, and IGFBP-3 from the blinded quality control samples were <11% for IGF-I, <6% for IGF-II, and <5% for IGFBP-3.

### Statistical analysis

We square root transformed the plasma biomarkers to improve normality and compared values for cases and controls using paired *t*-tests. Continuous and categorical covariates were compared using Wilcoxon signed rank, and *χ*^2^ tests, respectively. For the plasma biomarkers, all quartile cut-points were generated among the controls only and were determined separately for each prospective cohort. Spearman's correlation coefficients were calculated to examine the relationships among IGF-I, IGF-II, IGFBP-3, and selected covariates.

We computed odds ratios to estimate relative risks for the association of IGF-I, IGF-II, and IGFBP-3 and pancreatic cancer risk using conditional logistic regression. We also examined the molar ratio of IGF-I to IGFBP-3, as a possible surrogate for free IGF-I (for IGF-I, 1 ng ml^−1^=0.130 nM; and for IGFBP-3, 1 ng ml^−1^=0.036 nM). Tests for trend using two-sided *P*-values were calculated by entering the quartile-specific median values for IGF-1, IGF-II, IGFBP-3, and molar ratio of IGF-I and IGFBP-3 as continuous variables in logistic regression models. To confirm that data from the four cohorts could be combined, we utilized Cochran's Q to test for heterogeneity between cohorts.

We adjusted for covariates that were associated with pancreatic cancer risk in these cohorts, including body mass index (BMI, weight in kilograms/(height in meters)^2^), level of physical activity, history of diabetes mellitus, and history of regular multivitamin use. Body mass index and level of physical activity were included in models after division into quartiles. Other covariates, such as height, intake of vitamin D, intake of calcium, and total energy intake, were not included, as they were not consistently associated with pancreatic cancer risk across the cohorts.

Stratified analyses were conducted using unconditional logistic regression. We sequentially excluding cases and matched controls, with less than 4, 6, or 8 years between plasma collection and cancer diagnosis to evaluate whether the influence of IGF-I, IGF-II, or IGFBP-3 would change with longer follow-up. All statistical analyses were performed using the SAS 8.2 statistical package (SAS Institute, Cary, NC, USA) and all *P*-values are two sided.

## RESULTS

From the four cohorts, 212 cases of pancreatic cancer were identified in participants who had provided blood two or more years before cancer diagnosis. Based on the matching factors of year of birth, smoking status, prospective cohort, month of blood draw, and fasting status, 636 control participants were chosen. One control developed pancreatic cancer and was removed from the study, resulting in 212 cases and 635 controls available for analysis. No samples fell below the lowest concentration on the standard curves for the IGF-I, IGF-II, or IGFBP-3 assays. The comparison of IGF-I, IGF-II, and IGFBP-3 data from the four cohorts using Cochran's Q test for heterogeneity resulted in *P*-values of 0.58, 0.91, and 0.48, respectively, supporting the combined analysis of plasma marker data.

Baseline characteristics of the cases and matched controls are shown in Table 1. Participants who developed pancreatic cancer had a slightly higher BMI and were less likely to perform regular physical activity. No statistically significant differences were noted in other covariates or the plasma biomarkers. Spearman's correlation coefficients demonstrated a significant positive correlation of IGF-I with IGFBP-3, IGF-I : IGFBP-3 molar ratio, and height, and a significant inverse correlation with age. IGF-II was positively correlated with IGF-I and IGFBP-3, while little correlation was noted with the other covariates (correlation coefficients available in Table 1 of Supplementary Material).

Covariates related to the risk of pancreatic cancer with a *P*-value of less than 0.20 were included in multivariate analyses. Before and after adjustment for these covariates (BMI, level of physical activity, history of diabetes mellitus and regular multivitamin use), IGF-I, IGFBP-3, the IGF-I : IGFBP-3 molar ratio, and IGF-II were not associated with risk of pancreatic cancer (Table 2). The adjusted relative risk for the highest quartile of IGF-I compared with the lowest quartile was 0.94 (95% confidence interval (CI), 0.60–1.48). When the model was also adjusted for IGFBP-3, little change was noted in this relative risk. The adjusted relative risk for the highest quartile of IGFBP-3 compared with the lowest quartile was 1.21 (95% CI, 0.75–1.92), which changed minimally after inclusion of IGF-I in the multivariate model. In addition, the adjusted relative risks for the top *vs* the bottom quartiles of IGF-I : IGFBP-3 molar ratio and IGF-II were 0.84 (95% CI, 0.54–1.31) and 0.96 (95% CI, 0.61–1.52), respectively.

To evaluate more extreme levels of IGF-I, IGF-II, and IGFBP-3, we repeated the analyses after categorizing these plasma markers into deciles. As compared with participants in the lowest decile, participants in the highest decile of IGF-I, IGFBP-3, IGF-I : IGFBP-3 molar ratio, and IGF-II had adjusted relative risks of pancreatic cancer of 0.98 (95% CI, 0.50–1.93), 1.28 (95% CI, 0.65–2.52), 0.93 (95% CI, 0.46–1.89), and 1.14 (95% CI, 0.58–2.24), respectively. Additionally, we evaluated the combined effect of IGF-I and IGFBP-3 on pancreatic cancer risk by categorizing the plasma markers into tertiles and constructing a 3 × 3 table. The adjusted relative risk in the highest tertile of IGF-I and the lowest tertile of IGFBP-3 was 1.77 (95% CI, 0.25–12.70) compared with those participants in the lowest tertile of IGF-I and highest tertile of IGFBP-3. Only 3 cases and 14 controls were located in this extreme category of simultaneously high IGF-I and low IGFBP-3.

We found no evidence of an association between IGF-I and risk of pancreatic cancer in subgroups defined by categories of age, gender, BMI, smoking status, level of physical activity (Table 3), or regular multivitamin use. Similarly, no associations were noted in these subgroups for stratified analyses of IGFBP-3, IGF-I : IGFBP-3 molar ratio, and IGF-II (data not shown). Moreover, our results remained unchanged after excluding participants with a history of diabetes mellitus (data not shown).

To assess for a possible influence of the IGF axis after longer periods of follow-up and to rule out an effect of preclinical disease on IGF levels, we sequentially excluded cases and matched controls, requiring longer periods of time between plasma collection and pancreatic cancer diagnosis. No association was noted between IGF-I, IGFBP-3, IGF-I : IGFBP-3 molar ratio, or IGF-II and risk of pancreatic cancer when 2, 4, 6, or 8 years were required between plasma collection and cancer diagnosis (Table 4).

## DISCUSSION

In this prospective, nested case–control study, we found no evidence that the risk of pancreatic cancer was influenced by prediagnostic plasma levels of IGF-I, IGF-II, or IGFBP-3. No association was observed when these serum markers were analysed by comparing the top *vs* the bottom quartiles or when more extreme values were analysed by comparing the top *vs* the bottom deciles. In addition, no association was noted within selected subgroups or when longer follow-up was required between plasma collection and cancer diagnosis. The molar ratio of IGF-I and IGFBP-3 also was not associated with the development of pancreatic cancer.

Two smaller studies have evaluated the association of IGF-I and IGFBP-3 with pancreatic cancer risk (Lin *et al*, 2004; Stolzenberg-Solomon *et al*, 2004). A study of 93 pancreatic cancer cases from the Alpha-Tocopherol, *β*-Carotene (ATBC) Cancer Prevention Study of Finnish, male smokers found no association between IGF-I, IGFBP-3, or IGF-I : IGFBP-3 molar ratio and the risk of pancreatic cancer (Stolzenberg-Solomon *et al*, 2004). A study of 69 pancreatic cancer cases from the Japan Collaborative Cohort Study for Evaluation of Cancer Risk reported a nonsignificant increase in the risk of death from pancreatic cancer in the top *vs* the bottom quartile of IGF-I and IGFBP-3 (RR 2.31, 95% CI 0.70–7.64, and RR 2.53, 95% CI 0.93–6.85, respectively), which was attenuated when both plasma biomarkers were included in multivariate models (Lin *et al*, 2004). Thus, as in the current study, prior analyses of different patient populations have not supported a significant association between prediagnostic plasma levels of IGF-I or IGFBP-3 and the risk of pancreatic cancer.

Our study has several strengths, which lend further credibility to its negative findings. First, with 212 cases, our analysis includes more than the combined number of cases in the previous two prospective studies. Second, our cases are drawn from four different prospective cohort studies, increasing the generalizability of our results. Third, the collection of plasma and extensive exposure data before cancer diagnosis limits the potential influence of subclinical malignancy on our results. Finally, the ability to draw matched controls from a large pool of participants in each cohort study limits the potential for selection bias.

A limitation of our study is an inability to correlate the risk of pancreatic cancer with long-term levels of these plasma markers, since IGF-I, IGF-II, and IGFBP-3 were measured at a single point in time. However, previous evidence suggests that IGF levels are relatively stable over time in adults, such that a single measurement is a reasonable proxy for levels of long-term exposure (Goodman-Gruen and Barrett-Connor, 1997; Chan *et al*, 1998; Platz *et al*, 1999). Moreover, previous studies in these cohorts indicate that baseline plasma levels of IGF-I and IGFBP-3 are significantly associated with the risk of several other malignancies (Chan *et al*, 1998; Hankinson *et al*, 1998; Ma *et al*, 1999).

If a long latency period is required to see an effect of IGF-I, IGF-II, or IGFBP-3, then a second limitation of our study could be an inadequate duration of follow-up. However, the lack of association between IGF-I, IGF-II, or IGFBP-3 and pancreatic cancer in cases and matched controls with eight or more years between plasma collection and cancer diagnosis is reassuring that we are not missing a large effect of these plasma markers. In addition, the nested case–control study from the ATBC with up to 12.7 years between plasma collection and cancer diagnosis did not observe such an association (Stolzenberg-Solomon *et al*, 2004). Finally, our study measures plasma levels of IGF-I, IGF-II, and IGFBP-3 and not local tissue levels in the pancreas. Therefore, if circulating levels do not reflect local levels of these proteins, the observed odds ratios may not represent the true activity of IGF in the pancreas. Since circulating levels of IGF-I and IGFBP-3 have been correlated with the risk of several other malignancies (Renehan *et al*, 2004), our results do demonstrate that a similar association with pancreatic cancer risk is unlikely to be present in participants from our four cohorts.

Although tobacco use and increasing age are the best-defined risk factors for pancreatic cancer, several environmental and lifestyle factors have emerged that may be associated with this malignancy (Fuchs *et al*, 1996; Li *et al*, 2004). Studies have implicated obesity, lack of exercise, dietary glycemic load (an estimation of post-prandial glucose response), and diabetes mellitus as risk factors for pancreatic cancer (Gapstur *et al*, 2000; Michaud *et al*, 2001, 2002; Eberle *et al*, 2005; Huxley *et al*, 2005; Larsson *et al*, 2005). The mechanisms by which these factors increase cancer risk have been hypothesized to involve insulin and the insulin-like growth factor axis, which are intimately involved in glucose and energy homeostasis (Kaaks and Lukanova, 2001; Jerome *et al*, 2003).

Pancreatic ductal cells are exposed to insulin concentrations that are 20-fold higher than those present in the systemic circulation, due to the close proximity of these cells to the insulin-secreting cells of the islets of Langerhans (Hennig *et al*, 2004). Experimental studies have demonstrated that insulin may have growth-promoting effects on pancreatic ductal adenocarcinoma cells (Fisher *et al*, 1996; Wang *et al*, 1998; Ding *et al*, 2000) and that peripheral insulin resistance promotes pancreatic ductal carcinogenesis (Bell *et al*, 1988; Schneider *et al*, 2001). Additionally, treatment with metformin, an oral hypoglycemic agent that leads to decreases in peripheral insulin resistance and pancreatic insulin production, may prevent the development of malignant lesions (Schneider *et al*, 2001). These experimental studies support a biologic mechanism whereby elevated concentrations of serum insulin may promote the development of pancreatic ductal carcinoma. Further support for this mechanisms is lent by epidemiologic studies in which prediagnostic elevations in fasting serum glucose, post-load plasma glucose, and fasting serum insulin have been associated with an elevated risk of pancreatic cancer (Gapstur *et al*, 2000; Jee *et al*, 2005; Stolzenberg-Solomon *et al*, 2005).

Experimental data also support a role for the insulin-like growth factor axis in the pathogenesis of pancreatic cancer. *In vitro* studies have demonstrated that IGF-I and IGF-II are potent mitogens for cultured human pancreatic cancer cells (Ohmura *et al*, 1990; Bergmann *et al*, 1995; Stoeltzing *et al*, 2003; Zeng *et al*, 2003) and that IGF binding proteins can have opposing actions, in part by binding IGF-I and IGF-II (Rechler, 1997), but also by direct inhibitory effects on target cells (Rajah *et al*, 1997). The IGF axis also interacts with the insulin pathway, as elevated levels of insulin increase free, biologically active IGF-I, and alter concentrations of several IGF binding proteins (Giovannucci, 2003). For several malignancies, both elevated levels of circulating insulin and IGF-I have been associated with increased risk (Kaaks *et al*, 2000; Wei *et al*, 2005). Results from the current study and studies of insulin and insulin resistance suggest that pancreatic carcinogenesis may be influenced by circulating insulin levels, whereas plasma levels of IGF-I, IGF-II, and IGFBP-3 may have little effect on the long-term risk for this malignancy. But given the intimate relationship of multiple components of the IGF axis and insulin, the IGF axis may still play a role in the pathogenesis of pancreatic cancer that was not elucidated in the current study. Further studies of the interactions of insulin and the IGF axis in the pathogenesis of pancreatic cancer should help to advance our understanding of the mechanisms by which multiple anthropometric factors lead to an increased risk for this highly lethal malignancy.

## Figures and Tables

**Table 1:** Baseline characteristics of pancreatic cancer cases and matched controls

**Variable**	**Cases (*n*=212)**	**Controls (*n*=635)**	***P*-value^a^**
*Cohort*			Matched
HPFS	32	96	
PHS	57	171	
NHS	49	146	
WHI	74	222	
			
Age (years)	62.2±8.3	61.7±8.4	Matched
			
*Gender*			Matched
Female (%)	58	58	
Male (%)	42	42	
			
BMI (kg m^−2^)	26.6±4.8	26.1±5.2	0.05
Height (inches)	66.8±3.9	66.9±3.9	0.78
			
*Smoking status*			Matched
Never (%)	38	39	
Past (%)	43	45	
Current (%)	18	17	
			
*History of diabetes mellitus*			0.11
Yes (%)	7	4	
No (%)	93	96	
			
*Physical activity* b			0.05
Low (%)	60	53	
High (%)	40	47	
			
*History of regular multivitamin use*			0.10
Yes (%)	42	36	
No (%)	58	64	
			
Vitamin D intake (IU)^c^	400.5±276.8	387.4±274.3	0.62
Total energy intake (kcal)^c^	1675.4±554.8	1738.9±618.7	0.35
			
*Fasting status*			Matched
⩽8 h or unknown (%)	30	30	
>8 h (%)	70	70	
			
IGF-I (ng ml^−1^)	164.4±66.2	167.7±68.2	0.49
IGFBP-3 (ng ml^−1^)	4340.9±953.8	4325.6±872.5	0.92
IGF-1 : IGFBP-3 molar ratio	0.136±0.045	0.139±0.045	0.36
IGF-II (ng ml^−1^)	1058.7±256.0	1060.1±229.4	0.82

HPFS=Health Professionals Follow-up Study; PHS=Physicians' Health Study; NHS=Nurses' Health Study; WHI=Women's Health Initiative; BMI= body mass index; IGF-I=insulin-like growth factor-I; IGFBP-3=insulin-like growth factor binding protein-3; IGF-II=insulin-like growth factor-II.

Mean±standard deviation.

a IGF-I, IGFBP-3, IGF-I : IGFBP-3 molar ratio, and IGF-II were square root transformed to improve normality and analysed using paired *t*-tests. Covariates were analysed using Wilcoxon signed rank for continuous variables, and *χ*^2^ for categorical variables.

b Below (low) or above (high) the median level of physical activity.

c HPFS, NHS, and WHI participants.

**Table 2:** Relative risk (95% CI) of pancreatic cancer according to quartile of IGF-I, IGFBP-3, IGF-I : IGFBP-3 molar ratio, and IGF-II

	**Quartile 1**	**Quartile 2**	**Quartile 3**	**Quartile 4**	***P*-value, trend**
*IGF-I*					
Median (ng ml^−1^)	97.1	142.0	177.2	242.2	
Cases/controls	65/157	42/160	50/158	55/160	
RR^a^	1.0	0.63 (0.41–0.99)	0.78 (0.51–1.19)	0.83 (0.54–1.28)	0.57
RR^b^	1.0	0.67 (0.42–1.06)	0.84 (0.54–1.30)	0.94 (0.60–1.48)	0.97
RR^c^	1.0	0.62 (0.38–1.00)	0.73 (0.44–1.21)	0.78 (0.44–1.38)	0.56
					
*IGFBP-3*					
Median (ng ml^−1^)	3345.3	4043.6	4539.4	5290.2	
Cases/controls	51/158	53/158	50/158	58/161	
RR^a^	1.0	1.03 (0.66–1.62)	0.98 (0.63–1.54)	1.13 (0.72–1.77)	0.66
RR^b^	1.0	1.06 (0.67–1.69)	1.11 (0.70–1.76)	1.21 (0.75–1.92)	0.42
RR^d^	1.0	1.19 (0.73–1.95)	1.29 (0.76–2.19)	1.38 (0.76–2.51)	0.28
					
*IGF-I : IGFBP-3 molar ratio*					
Median	0.09	0.11	0.13	0.19	
Cases/controls	68/157	41/160	50/158	53/160	
RR^a^	1.0	0.60 (0.38–0.93)	0.74 (0.48–1.12)	0.77 (0.50–1.19)	0.49
RR^b^	1.0	0.62 (0.39–0.97)	0.79 (0.51–1.22)	0.84 (0.54–1.31)	0.80
					
*IGF-II*					
Median (ng ml^−1^)	802.8	986.2	1116.7	1335.0	
Cases/controls	56/161	47/158	59/159	50/157	
RR^a^	1.0	0.86 (0.55–1.33)	1.06 (0.70–1.62)	0.91 (0.59–1.42)	0.88
RR^b^	1.0	0.89 (0.57–1.40)	1.12 (0.72–1.73)	0.96 (0.61–1.52)	0.93

CI=confidence interval; IGF-I=insulin-like growth factor-I; IGFBP-3=insulin-like growth factor binding protein-3; IGF-II=insulin-like growth factor-II.

a Matched for year of birth, smoking status, fasting status, month of blood draw, and prospective cohort.

b Matched for year of birth, smoking status, fasting status, month of blood draw and prospective cohort, and adjusted for BMI, regular multivitamin use, level of physical activity, and history of diabetes.

c Matched for year of birth, smoking status, fasting status, month of blood draw and prospective cohort, and adjusted for BMI, regular multivitamin use, level of physical activity, history of diabetes, and IGFBP-3.

d Matched for year of birth, smoking status, fasting status, month of blood draw and prospective cohort, and adjusted for BMI, regular multivitamin use, level of physical activity, history of diabetes, and IGF-I.

**Table 3:** Relative risk of pancreatic cancer according to quartile of insulin-like growth factor-I (IGF-I) in subgroups defined by selected variables

**Covariate**	**Cases/controls**	**Quartile 1**	**Quartile 2**	**Quartile 3**	**Quartile 4**	**95 % CI^a^ of Quartile 4**	***P*-value, trend**
*Age (years)* b							
⩽62	102/335	1.0	0.74	0.86	0.92	0.48–1.78	0.98
>62	110/300	1.0	0.59	0.87	1.00	0.54–1.88	0.83
							
*Gender*							
Male	89/267	1.0	0.75	0.80	1.40	0.69–2.84	0.29
Female	123/368	1.0	0.63	0.86	0.76	0.42–1.38	0.53
							
*BMI (kg m* ^*−2*^ *)*							
<25	88/306	1.0	0.88	0.87	0.75	0.37–1.51	0.43
⩾25	124/329	1.0	0.59	0.81	1.17	0.65–2.11	0.42
							
*Smoking status*							
Never	81/243	1.0	0.78	0.77	1.00	0.47–2.13	0.97
Past	92/283	1.0	0.75	0.83	1.06	0.54–2.10	0.81
Current	39/107	1.0	0.38	0.85	0.69	0.22–2.16	0.79
							
*Level of activity* c							
Low	128/334	1.0	0.62	0.86	0.96	0.52–1.76	0.89
High	84/301	1.0	0.71	0.63	0.86	0.44–1.69	0.73
							
*Multivitamin use*							
No	122/408	1.0	0.84	0.88	0.88	0.49–1.59	0.74
Yes	90/227	1.0	0.53	0.86	1.08	0.53–2.21	0.62
							
*IGFBP-3* d							
Low	104/316	1.0	0.44	0.82	0.91	0.36–2.28	0.43
High	108/319	1.0	1.26	0.95	1.19	0.48–2.97	0.74

CI=confidence interval; BMI=body mass index; IGFBP-3=insulin-like growth factor binding protein-3.

Multivariate relative risks adjusted for year of birth, smoking status, fasting status, prospective cohort, BMI, level of physical activity, regular multivitamin use, and history of diabetes mellitus. In each stratified analysis, the stratification variable was excluded from the model.

a Ninety-five percent confidence interval.

b Sixty-two years of age is the median age for controls.

c High is above the median level, while low is below the median level of physical activity.

d High is above the median level, while low is below the median level of IGFBP-3.

**Table 4:** Relative risk of pancreatic cancer according to time from plasma collection to pancreatic cancer diagnosis by quartiles of IGF-I, IGFBP-3, IGF-I : IGFBP-3 molar ratio, and IGF-II^a^

	**Quartile 1**	**Quartile 2**	**Quartile 3**	**Quartile 4**	**95% CI^a^ for Quartile 4**	***P*-value, trend**
*IGF-I (years)*						
⩾2	1.0	0.67	0.82	0.95	0.61–1.46	0.96
⩾4	1.0	0.68	1.03	0.73	0.43–1.25	0.45
⩾6	1.0	0.73	1.02	0.69	0.35–1.37	0.42
⩾8	1.0	0.74	1.12	0.79	0.35–1.81	0.75
						
*IGFBP-3 (years)*						
⩾2	1.0	1.08	1.08	1.27	0.82–1.98	0.29
⩾4	1.0	0.67	0.92	1.06	0.64–1.74	0.64
⩾6	1.0	0.53	1.05	0.88	0.47–1.67	0.96
⩾8	1.0	0.46	0.83	0.86	0.40–1.86	0.94
						
*IGF-I : IGFBP-3 molar ratio (years)*						
⩾2	1.0	0.64	0.77	0.85	0.55–1.32	0.82
⩾4	1.0	0.73	0.83	0.73	0.43–1.23	0.34
⩾6	1.0	0.67	0.70	0.83	0.44–1.58	0.78
⩾8	1.0	0.80	0.62	1.03	0.47–2.22	0.82
						
*IGF-II (years)*						
⩾2	1.0	0.86	1.07	1.00	0.64–1.57	0.81
⩾4	1.0	0.65	0.75	0.94	0.57–1.55	0.90
⩾6	1.0	0.62	0.74	0.85	0.45–1.60	0.68
⩾8	1.0	0.58	0.76	0.71	0.33–1.55	0.46

CI=confidence interval; IGF-I=insulin-like growth factor-I, IGFBP-3=insulin-like growth factor binding protein-3, IGF-II=insulin-like growth factor-II.

Multivariate relative risks are adjusted for year of birth, smoking status, fasting status, prospective cohort, regular multivitamin use, level of physical activity, history of diabetes mellitus, and BMI.

a Ninety-five percent CI.
