# Supplementary material for: Circulating insulin-like growth factor axis and the risk of pancreatic cancer in four prospective cohorts
Source: Br J Cancer. 2007 May 29;97(1):98–104. doi: 10.1038/sj.bjc.6603826 (PMC2359655; doi:10.1038/sj.bjc.6603826)
Supplement: Supplemental Material [file 6603826x1.doc]

Supplemental Material

Table 1. Spearman correlation coefficients between components of the insulin-like growth factor axis and covariates among matched pancreatic cancer controls

| Variable^†^ | IGF-I | IGFBP-3 | Molar ratio | IGF-II | Age | BMI | Physical activity | Height |
| --- | --- | --- | --- | --- | --- | --- | --- | --- |
| IGF-I | 1.0 |  |  |  |  |  |  |  |
| IGFBP-3 | 0.54* | 1.0 |  |  |  |  |  |  |
| Molar ratio | 0.85* | 0.06 | 1.0 |  |  |  |  |  |
| IGF-II | 0.48* | 0.87* | 0.06 | 1.0 |  |  |  |  |
| Age | -0.35* | -0.09^‡^ | -0.36* | -0.07 | 1.0 |  |  |  |
| BMI | -0.10^‡^ | 0.03 | -0.13* | 0.07 | 0.08^‡^ | 1.0 |  |  |
| Physical activity | -0.01 | 0.09^‡^ | -0.05 | 0.06 | 0.09^‡^ | -0.06 | 1.0 |  |
| Height | 0.26* | -0.15* | 0.40* | -0.04 | -0.35* | -0.12^‡^ | -0.07 | 1.0 |
| Total energy intake | 0.12^‡^ | 0.01 | 0.14^‡^ | -0.03 | -0.15^‡^ | -0.04 | 0.12^‡^ | 0.16* |

^†^ IGF-I = insulin-like growth factor-I, IGFBP-3 = insulin-like growth factor binding protein-3, molar ratio = IGF-I:IGFBP-3 molar ratio, IGF-II = insulin-like growth factor-II, BMI = body-mass index

^‡^ p<0.05

* p<0.001
